# 2-(4-Methyl­phen­yl)benzonitrile

**DOI:** 10.1107/S160053681101988X

**Published:** 2011-06-11

**Authors:** M. S. Siddegowda, Jerry P. Jasinski, James A. Golen, H. S. Yathirajan

**Affiliations:** a Department of Studies in Chemistry, University of Mysore, Manasagangotri, Mysore 570 006, India; b Department of Chemistry, Keene State College, 229 Main Street, Keene, NH 03435-2001, USA

## Abstract

In the title compound, C_14_H_11_N, the dihedral angle between the mean planes of the two benzene rings is 44.6 (7)°. The crystal packing is stabilized by weak inter­molecular π–π stacking inter­actions, the centroid–centroid distances being 3.8172 (12) and 3.9349 (12) Å.

## Related literature

For the synthesis of pharmaceutically active compounds, see: Gillis & Markham (1997 ▶); Markham & Goa (1997 ▶). For related structures, see: Gerkin (1998 ▶); Narasegowda *et al.* (2005 ▶); Yathirajan *et al.* (2005 ▶). For standard bond lengths, see Allen *et al.* (1987 ▶).
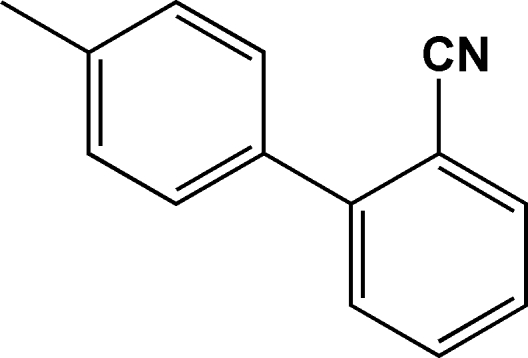


## Experimental

### 

#### Crystal data

C_14_H_11_N
*M* *_r_* = 193.24
Orthorhombic, 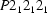

*a* = 7.6726 (4) Å
*b* = 11.4037 (5) Å
*c* = 12.2426 (5) Å
*V* = 1071.18 (9) Å^3^
*Z* = 4
Mo *K*α radiation
μ = 0.07 mm^−1^
*T* = 173 K
0.30 × 0.25 × 0.20 mm

#### Data collection

Oxford Diffraction Xcalibur Eos Gemini diffractometer
Absorption correction: multi-scan (*CrysAlis RED*; Oxford Diffraction, 2010 ▶) *T*_min_ = 0.979, *T*_max_ = 0.986
3786 measured reflections
1546 independent reflections
1322 reflections with *I* > 2σ(*I*)
*R*_int_ = 0.017

#### Refinement

*R*[*F* ^2^ > 2σ(*F* ^2^)] = 0.040
*wR*(*F* ^2^) = 0.105
*S* = 1.07
1546 reflections
137 parameters
H-atom parameters constrained
Δρ_max_ = 0.22 e Å^−3^
Δρ_min_ = −0.14 e Å^−3^

### 

Data collection: *CrysAlis PRO* (Oxford Diffraction, 2010 ▶); cell refinement: *CrysAlis PRO*; data reduction: *CrysAlis RED* (Oxford Diffraction, 2010 ▶); program(s) used to solve structure: *SHELXS97* (Sheldrick, 2008 ▶); program(s) used to refine structure: *SHELXL97* (Sheldrick, 2008 ▶); molecular graphics: *SHELXTL* (Sheldrick, 2008 ▶); software used to prepare material for publication: *SHELXTL*.

## Supplementary Material

Crystal structure: contains datablock(s) global, I. DOI: 10.1107/S160053681101988X/xu5222sup1.cif

Structure factors: contains datablock(s) I. DOI: 10.1107/S160053681101988X/xu5222Isup2.hkl

Supplementary material file. DOI: 10.1107/S160053681101988X/xu5222Isup3.cml

Additional supplementary materials:  crystallographic information; 3D view; checkCIF report

## Figures and Tables

**Fig. 1.**
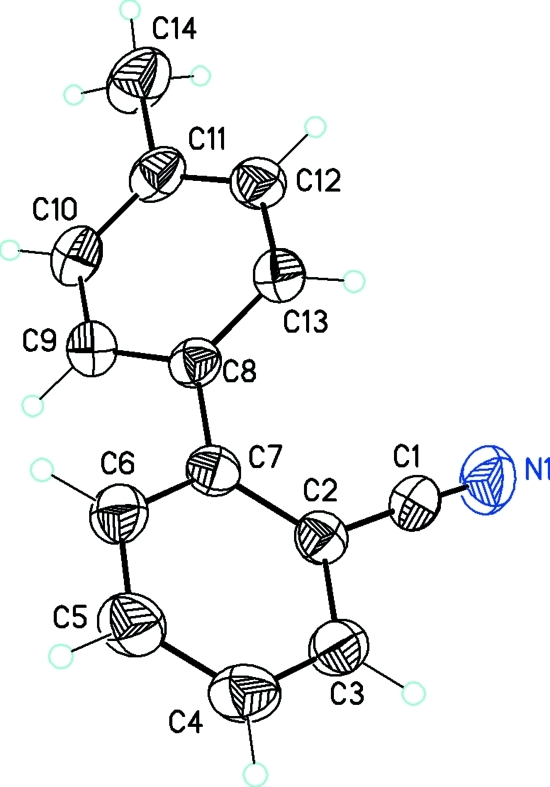
Molecular structure of the title compound showing the atom labeling scheme and 50% probability displacement ellipsoids.

**Fig. 2.**
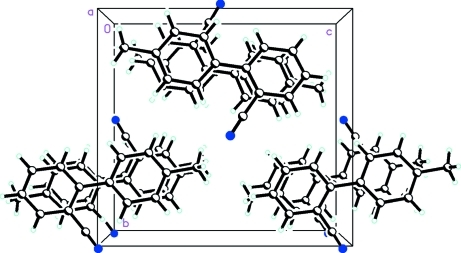
Packing diagram of the title compound viewed down the a axis.

## supplementary crystallographic information

### Comment

The title compound, C_14_H_11_N, (I), is used to synthesize various biologically active and pharmaceutical compounds viz., losartan, valsartan, candesartan, etc. (Gillis & Markham, 1997; Markham & Goa, 1997).  The crystal structures of 4,4'-dimethylbiphenyl-2,2'-dicarboxylic acid (Gerkin, 1998), 4'-methylbiphenyl-2-carboxylic acid (Narasegowda *et al.*, 2005) and 4'-(2-butyl-4-chloro-5-formylimidazol-1-ylmethyl)biphenyl-2-carbonitrile (Yathirajan *et al.*, 2005) have been reported. In view of its importance in order to determine the conformation of this molecule, a crystal structure determination of (I) is reported.

In the title compound, C_14_H_11_N,, the dihedral angle between the mean planes of the two benzene rings is 44.6 (7)° (Fig. 1). Bond lengths and angles are in normal positions (Allen *et al.*, 1987). Crystal packing is stabilized by weak π-π stacking interactions (Fig. 2, Table 1).

### Experimental

The title compound was obtained as a gift sample from R. L. Fine Chem, Bangalore. X-ray quality crystals were obtained by slow evaporation of methanol solution (m.p.: 323-325 K).

### Refinement

All of the H atoms were placed in their calculated positions and then refined using the riding model with C—H = 0.95 Å (aromatic) or 0.98 Å (CH_3_).  Isotropic displacement parameters for these atoms were set to 1.19-1.21 (aromatic) or 1.50 (CH_3_) times *U*_eq_ of the parent atom.

### Crystal data

**Table:**

C_14_H_11_N	*F*(000) = 408
*M**_r_* = 193.24	*D*_x_ = 1.198 Mg m^−^^3^
Orthorhombic, *P*2_1_2_1_2_1_	Mo *K*α radiation, λ = 0.71073 Å
Hall symbol: P 2ac 2ab	Cell parameters from 2201 reflections
*a* = 7.6726 (4) Å	θ = 3.3–32.3°
*b* = 11.4037 (5) Å	µ = 0.07 mm^−^^1^
*c* = 12.2426 (5) Å	*T* = 173 K
*V* = 1071.18 (9) Å^3^	Block, colorless
*Z* = 4	0.30 × 0.25 × 0.20 mm

### Data collection

**Table:**

Oxford Diffraction Xcalibur Eos Gemini diffractometer	1546 independent reflections
Radiation source: Enhance (Mo) X-ray Source	1322 reflections with *I* > 2σ(*I*)
graphite	*R*_int_ = 0.017
Detector resolution: 16.1500 pixels mm^-1^	θ_max_ = 28.3°, θ_min_ = 3.3°
ω scans	*h* = −10→10
Absorption correction: multi-scan (*CrysAlis RED*; Oxford Diffraction, 2010)	*k* = −7→15
*T*_min_ = 0.979, *T*_max_ = 0.986	*l* = −16→16
3786 measured reflections	

### Refinement

**Table:**

Refinement on *F*^2^	Primary atom site location: structure-invariant direct methods
Least-squares matrix: full	Secondary atom site location: difference Fourier map
*R*[*F*^2^ > 2σ(*F*^2^)] = 0.040	Hydrogen site location: inferred from neighbouring sites
*wR*(*F*^2^) = 0.105	H-atom parameters constrained
*S* = 1.07	*w* = 1/[σ^2^(*F*_o_^2^) + (0.0516*P*)^2^ + 0.1272*P*] where *P* = (*F*_o_^2^ + 2*F*_c_^2^)/3
1546 reflections	(Δ/σ)_max_ = 0.008
137 parameters	Δρ_max_ = 0.22 e Å^−^^3^
0 restraints	Δρ_min_ = −0.14 e Å^−^^3^

### Special details

**Table:**

Geometry. All esds (except the esd in the dihedral angle between two l.s. planes) are estimated using the full covariance matrix. The cell esds are taken into account individually in the estimation of esds in distances, angles and torsion angles; correlations between esds in cell parameters are only used when they are defined by crystal symmetry. An approximate (isotropic) treatment of cell esds is used for estimating esds involving l.s. planes.
Refinement. Refinement of *F*^2^ against ALL reflections. The weighted *R*-factor *wR* and goodness of fit *S* are based on *F*^2^, conventional *R*-factors *R* are based on *F*, with *F* set to zero for negative *F*^2^. The threshold expression of *F*^2^ > σ(*F*^2^) is used only for calculating *R*-factors(gt) *etc*. and is not relevant to the choice of reflections for refinement. *R*-factors based on *F*^2^ are statistically about twice as large as those based on *F*, and *R*- factors based on ALL data will be even larger.

### Fractional atomic coordinates and isotropic or equivalent isotropic displacement parameters (Å^2^)

**Table:**

	*x*	*y*	*z*	*U*_iso_*/*U*_eq_	
N1	0.2954 (3)	0.53397 (15)	0.51932 (16)	0.0599 (5)	
C1	0.2409 (3)	0.45620 (16)	0.56640 (15)	0.0403 (4)	
C2	0.1730 (2)	0.36058 (15)	0.62994 (15)	0.0350 (4)	
C3	0.1706 (3)	0.37476 (16)	0.74343 (15)	0.0413 (4)	
H3A	0.2117	0.4456	0.7751	0.050*	
C4	0.1091 (3)	0.28649 (19)	0.80918 (16)	0.0484 (5)	
H4A	0.1060	0.2963	0.8862	0.058*	
C5	0.0518 (3)	0.1835 (2)	0.76255 (16)	0.0498 (5)	
H5A	0.0090	0.1223	0.8079	0.060*	
C6	0.0559 (3)	0.16831 (18)	0.65050 (16)	0.0427 (5)	
H6A	0.0171	0.0962	0.6202	0.051*	
C7	0.1155 (2)	0.25620 (15)	0.58127 (13)	0.0331 (4)	
C8	0.1185 (2)	0.23705 (15)	0.46123 (13)	0.0333 (4)	
C9	0.1780 (3)	0.13095 (16)	0.41890 (16)	0.0412 (4)	
H9A	0.2178	0.0715	0.4672	0.049*	
C10	0.1798 (3)	0.11109 (17)	0.30733 (16)	0.0459 (5)	
H10A	0.2211	0.0381	0.2804	0.055*	
C11	0.1230 (3)	0.19495 (18)	0.23447 (15)	0.0441 (5)	
C12	0.0627 (3)	0.30048 (18)	0.27609 (16)	0.0432 (5)	
H12A	0.0224	0.3595	0.2275	0.052*	
C13	0.0604 (2)	0.32112 (16)	0.38739 (15)	0.0383 (4)	
H13A	0.0183	0.3941	0.4139	0.046*	
C14	0.1238 (4)	0.1727 (2)	0.11270 (17)	0.0671 (7)	
H14A	0.1788	0.2391	0.0753	0.101*	
H14B	0.0038	0.1637	0.0867	0.101*	
H14C	0.1895	0.1009	0.0972	0.101*	

### Atomic displacement parameters (Å^2^)

**Table:**

	*U*^11^	*U*^22^	*U*^33^	*U*^12^	*U*^13^	*U*^23^
N1	0.0834 (14)	0.0413 (9)	0.0550 (10)	−0.0116 (10)	0.0026 (11)	0.0037 (8)
C1	0.0460 (10)	0.0356 (8)	0.0393 (9)	0.0018 (9)	−0.0010 (9)	−0.0051 (8)
C2	0.0344 (9)	0.0356 (8)	0.0349 (8)	0.0027 (8)	0.0011 (7)	0.0004 (7)
C3	0.0423 (10)	0.0424 (9)	0.0391 (10)	0.0009 (9)	−0.0028 (8)	−0.0043 (8)
C4	0.0542 (12)	0.0583 (12)	0.0328 (8)	−0.0027 (11)	0.0002 (9)	0.0020 (9)
C5	0.0559 (12)	0.0539 (11)	0.0395 (9)	−0.0100 (11)	0.0019 (9)	0.0107 (9)
C6	0.0454 (10)	0.0414 (9)	0.0414 (10)	−0.0099 (9)	−0.0005 (8)	0.0031 (8)
C7	0.0298 (8)	0.0367 (8)	0.0329 (8)	0.0023 (8)	−0.0003 (7)	0.0009 (7)
C8	0.0303 (8)	0.0358 (8)	0.0337 (8)	−0.0045 (7)	−0.0004 (7)	0.0000 (7)
C9	0.0448 (10)	0.0347 (9)	0.0441 (10)	−0.0005 (8)	−0.0020 (9)	0.0007 (8)
C10	0.0526 (11)	0.0380 (9)	0.0472 (11)	−0.0031 (9)	0.0032 (10)	−0.0092 (8)
C11	0.0489 (11)	0.0468 (10)	0.0366 (8)	−0.0151 (10)	0.0036 (9)	−0.0039 (8)
C12	0.0477 (11)	0.0449 (10)	0.0370 (8)	−0.0057 (9)	−0.0049 (8)	0.0056 (8)
C13	0.0391 (9)	0.0359 (8)	0.0397 (9)	0.0017 (8)	−0.0019 (8)	−0.0009 (8)
C14	0.0991 (19)	0.0644 (13)	0.0378 (10)	−0.0186 (16)	0.0036 (13)	−0.0095 (11)

### Geometric parameters (Å, °)

**Table:**

N1—C1	1.137 (2)	C8—C9	1.393 (2)
C1—C2	1.437 (2)	C9—C10	1.385 (3)
C2—C3	1.399 (2)	C9—H9A	0.9500
C2—C7	1.402 (2)	C10—C11	1.378 (3)
C3—C4	1.373 (3)	C10—H10A	0.9500
C3—H3A	0.9500	C11—C12	1.386 (3)
C4—C5	1.378 (3)	C11—C14	1.512 (3)
C4—H4A	0.9500	C12—C13	1.383 (3)
C5—C6	1.383 (3)	C12—H12A	0.9500
C5—H5A	0.9500	C13—H13A	0.9500
C6—C7	1.390 (2)	C14—H14A	0.9800
C6—H6A	0.9500	C14—H14B	0.9800
C7—C8	1.486 (2)	C14—H14C	0.9800
C8—C13	1.391 (2)		
			
N1—C1—C2	177.67 (19)	C10—C9—C8	120.81 (17)
C3—C2—C7	121.07 (17)	C10—C9—H9A	119.6
C3—C2—C1	117.04 (16)	C8—C9—H9A	119.6
C7—C2—C1	121.87 (15)	C11—C10—C9	121.45 (18)
C4—C3—C2	120.14 (18)	C11—C10—H10A	119.3
C4—C3—H3A	119.9	C9—C10—H10A	119.3
C2—C3—H3A	119.9	C10—C11—C12	118.03 (17)
C3—C4—C5	119.46 (17)	C10—C11—C14	121.3 (2)
C3—C4—H4A	120.3	C12—C11—C14	120.6 (2)
C5—C4—H4A	120.3	C13—C12—C11	120.95 (18)
C4—C5—C6	120.7 (2)	C13—C12—H12A	119.5
C4—C5—H5A	119.6	C11—C12—H12A	119.5
C6—C5—H5A	119.6	C12—C13—C8	121.25 (17)
C5—C6—C7	121.45 (19)	C12—C13—H13A	119.4
C5—C6—H6A	119.3	C8—C13—H13A	119.4
C7—C6—H6A	119.3	C11—C14—H14A	109.5
C6—C7—C2	117.15 (15)	C11—C14—H14B	109.5
C6—C7—C8	120.14 (16)	H14A—C14—H14B	109.5
C2—C7—C8	122.70 (15)	C11—C14—H14C	109.5
C13—C8—C9	117.51 (16)	H14A—C14—H14C	109.5
C13—C8—C7	122.45 (16)	H14B—C14—H14C	109.5
C9—C8—C7	120.03 (16)		
			
C7—C2—C3—C4	−1.0 (3)	C6—C7—C8—C9	−43.5 (2)
C1—C2—C3—C4	−179.36 (18)	C2—C7—C8—C9	135.8 (2)
C2—C3—C4—C5	0.8 (3)	C13—C8—C9—C10	0.4 (3)
C3—C4—C5—C6	0.1 (3)	C7—C8—C9—C10	179.27 (18)
C4—C5—C6—C7	−0.8 (3)	C8—C9—C10—C11	−0.1 (3)
C5—C6—C7—C2	0.6 (3)	C9—C10—C11—C12	−0.3 (3)
C5—C6—C7—C8	179.9 (2)	C9—C10—C11—C14	−179.5 (2)
C3—C2—C7—C6	0.3 (3)	C10—C11—C12—C13	0.3 (3)
C1—C2—C7—C6	178.58 (18)	C14—C11—C12—C13	179.5 (2)
C3—C2—C7—C8	−179.05 (18)	C11—C12—C13—C8	0.1 (3)
C1—C2—C7—C8	−0.7 (3)	C9—C8—C13—C12	−0.5 (3)
C6—C7—C8—C13	135.2 (2)	C7—C8—C13—C12	−179.24 (17)
C2—C7—C8—C13	−45.5 (3)		

### Table 1 Selected geometric parmeters (Å): π-π stacking interactions, Cg1 and Cg2 are the centroids of rings C2—C7 and C8—C13. Symmetry codes: (i) -1/2+x, 1/2-y, 1-z; (ii) 1/2+x, 1/2-y, 1-z.

**Table:**

*Cg*I···*Cg*J	Cg···Cg (Å)	CgI Perp (Å)	Cgj Perp (Å)
Cg1···Cg2^i^	3.8172 (12)	3.5763 (8)	-3.5789 (8)
Cg2···Cg1^ii^	3.9349 (12)	-3.5230 (8)	3.5154 (8)
